# Wolfram Syndrome in the Japanese Population; Molecular Analysis of *WFS1* Gene and Characterization of Clinical Features

**DOI:** 10.1371/journal.pone.0106906

**Published:** 2014-09-11

**Authors:** Kimie Matsunaga, Katsuya Tanabe, Hiroshi Inoue, Shigeru Okuya, Yasuharu Ohta, Masaru Akiyama, Akihiko Taguchi, Yukari Kora, Naoko Okayama, Yuichiro Yamada, Yasuhiko Wada, Shin Amemiya, Shigetaka Sugihara, Yuzo Nakao, Yoshitomo Oka, Yukio Tanizawa

**Affiliations:** 1 Division of Endocrinology, Metabolism, Hematological Science and Therapeutics, Yamaguchi University Graduate School of Medicine, Ube, Yamaguchi, Japan; 2 Division of Laboratory Yamaguchi University Hospital, Ube, Yamaguchi, Japan; 3 Department of Endocrinology, Diabetes, Geriatric Medicine, Akita University Graduate School of Medicine, Akita, Japan; 4 Department of Health and Nutrition, Kochi University, Kochi, Kochi Japan; 5 Department of Pediatrics, Saitama Medical University, Moroyama, Saitama, Japan; 6 Department of Pediatrics, Tokyo Women’s Medical University Medical Center East, Tokyo, Japan; 7 Division of Ophthalmology, Kinki University Sakai Hospital, Sakai, Osaka, Japan; 8 Division of Molecular Metabolism and Diabetes, Tohoku University Graduate School of medicine, Sendai, Miyagi, Japan; Graduate School of Medicine, University of the Ryukyus, Japan

## Abstract

**Background:**

Wolfram syndrome (WFS) is a recessive neurologic and endocrinologic degenerative disorder, and is also known as DIDMOAD (Diabetes Insipidus, early-onset Diabetes Mellitus, progressive Optic Atrophy and Deafness) syndrome. Most affected individuals carry recessive mutations in the Wolfram syndrome 1 gene (*WFS1*). However, the phenotypic pleiomorphism, rarity and molecular complexity of this disease complicate our efforts to understand WFS. To address this limitation, we aimed to describe complications and to elucidate the contributions of *WFS1* mutations to clinical manifestations in Japanese patients with WFS.

**Methodology:**

The minimal ascertainment criterion for diagnosing WFS was having both early onset diabetes mellitus and bilateral optic atrophy. Genetic analysis for *WFS1* was performed by direct sequencing.

**Principal Findings:**

Sixty-seven patients were identified nationally for a prevalence of one per 710,000, with 33 patients (49%) having all 4 components of DIDMOAD. In 40 subjects who agreed to participate in this investigation from 30 unrelated families, the earliest manifestation was DM at a median age of 8.7 years, followed by OA at a median age of 15.8 years. However, either OA or DI was the first diagnosed feature in 6 subjects. In 10, features other than DM predated OA. Twenty-seven patients (67.5%) had a broad spectrum of recessive mutations in *WFS1*. Two patients had mutations in only one allele. Eleven patients (27.5%) had intact *WFS1* alleles. Ages at onset of both DM and OA in patients with recessive *WFS1* mutations were indistinguishable from those in patients without *WFS1* mutations. In the patients with predicted complete loss-of-function mutations, ages at the onsets of both DM and OA were significantly earlier than those in patients with predicted partial-loss-of function mutations.

**Conclusion/Significance:**

This study emphasizes the clinical and genetic heterogeneity in patients with WFS. Genotype-phenotype correlations may exist in patients with *WFS1* mutations, as demonstrated by the disease onset.

## Introduction

Wolfram syndrome (WFS:OMIM 222300) was first described by Wolfram and Wagener in 1938 as the association of childhood-onset diabetes and optic atrophy [1]. It is now recognized as a recessive neurologic and endocrinologic degenerative disorder defined by the association of early onset insulin dependent diabetes mellitus and progressive bilateral optic atrophy [2]. Affected individuals may also have other clinical manifestations, particularly diabetes insipidus and sensory nerve deafness such that this disease is sometimes referred as DIDMOAD (diabetes insipidus, diabetes mellitus, optic atrophy, and deafness) syndrome. Because WFS is a progressive disorder, affected individuals experience a wide spectrum of symptoms during their lifetimes [3]. The lifespans of affected individuals are generally shortened as a consequence of neuropsychiatric problems, such as central respiratory failure, food aspiration and suicide [4]. Currently, no therapeutic intervention is known to alter the progression or the life expectancy of affected individuals.

A number of loss-of-function mutations of the Wolfram syndrome 1 gene (*WFS1*) have been described in patients with WFS [4], [5], [6]. *Wfs1* deficient mice have glucose intolerance associated with loss of pancreatic beta cells [7], [8], [9]. The gene product of *WFS1* (WFS1) is an endoplasmic reticulum (ER) embedded protein, which has been implicated in various cellular functions such as insulin secretion [7], [10] and processing [10], cell cycle regulation [11], [12], the unfolded protein response [9], [13] and cAMP production [14]. On the other hand, *WFS1* mutation was not identified in some patients, providing evidence of genetic heterogeneity for this disease. In fact, a specific mutation in a second gene (*WFS2*), also known as *CISD2*, has been described in affected Jordanian families [15].

Whereas there are numerous reports describing the features of WFS based on retrospective observations, the use of different diagnostic criteria and a different focus on clinical details of this syndrome complicate determination of the natural history of WFS [6], [16], [17], [18], [19], [20]. In addition, the wide spectrum of clinical symptoms and the molecular complexity of this disease hamper precise elucidation of any genotype-phenotype correlations.

This study is a cross-sectional case finding analysis of WFS in Japanese patients. Our objectives are to describe complications and prevalence, as well as to evaluate the contribution of *WFS1* mutations to clinical manifestations of this syndrome. Sixty-seven patients with WFS were recruited nationally. Among them, 40 were screened for *WFS1* mutations. We thus report both clinical and genetic features of Japanese patients with WFS.

## Patients and Methods

### Overview

Patients diagnosed with WFS over last 15-year were recruited nationally from diabetes referral centers, as well as pediatric and adult diabetes clinics by sending out a questionnaire including standardized information encompassing major clinical components known to be associated with WFS. Minimum ascertainment criteria for identification of patients were the coincident occurrence of insulin requiring diabetes mellitus with onset before age 30 years and bilateral optic atrophy with insidious onset and/or genetic confirmation of a *WFS1* mutation prior to this study. Diabetes mellitus was assessed by a physician or a pediatrician, and all subjects received ophthalmologic examinations by an experienced ophthalmologist. Other clinical features were assessed by either a physician or a specialist. Genetic analyses were performed after obtaining the subject’s consent.

### Ethics statement

The protocols for the clinical and genetic studies were approved by the ethics committee of Yamaguchi University Graduate School of Medicine. Subjects provided written informed consent themselves if they were adults. Minor children agreed to participate, after receiving an explanation, and their parents provided written consent. The subject identification numbers shown in this report are based on numbers in the registry for this study and are therefore not necessarily consecutive. Unrelated non-diabetic Japanese subjects ranging in age from 20 to 65 years had donated blood samples for DNA extraction after providing written consent for the genetic analyses under the approval of the ethics committee of Yamaguchi University Graduate School of Medicine.

### Estimation of prevalence

We estimated the prevalence of WFS in Japan by estimating all living patients who satisfied the ascertainment criteria in the Tokyo metropolitan area, Osaka metropolitan area and Tohoku region, Japan, in 2010. The major referral sources were physicians specializing in diabetes and pediatric endocrinologists. To assess the completeness of patient recruitment in this study, we identified 56 independent Japanese WFS cases reported since 1992 by searching the Pubmed and Japan Medical Abstracts Society (http://login.jamas.or.jp/) databases. Among these, 40 cases (71.4%) had been enrolled in the current study. Based on the assumption that the referral source might not have been able to identify all cases, we divided the number of living patients enrolled in this study by 0.714 as a correction to the estimated number of cases in each region.

## Genetic analysis

### Mutation analysis

Genomic DNA was extracted from peripheral blood mononuclear cells according to standard procedures. All *WFS1* exons, including intron/exon boundaries, 113 bp upstream from exon 1 and 51 bp from the 3′-untranslated region were amplified from genomic DNA by PCR using the primers shown in Table S1in File S1. Both strands were sequenced on an automated sequencer (ABI 3730, Applied Biosystems, Foster City, CA, USA). Results were compared to the *WFS1* genomic sequence (NT_006051.11), using the ATGC sequence assembling software (GENETYX, Tokyo, Japan).

After identification of unreported *WFS1* mutations which cause a single amino acid substitution or a single amino acid deletion, we genotyped each such mutation in duplicate in all studied individuals, using the TaqMan assay (Applied Biosystems) with the DNA probes described in Table S2 in File S1. There was 100% concordance between sequencing and genotyping results. At least 100 unrelated Japanese controls were screened for these mutations employing the same TaqMan assay. We also screened 10 affected individuals for mitochondrial DNA deletions and point mutations (m.3243A>G tRNAleu; OMIM #590050) and Leber Hereditary Optic Atrophy (LHON; OMIM #535000) associated mutations: m.11778G>A by PCR-RFLP (SRL corporation, Tokyo, Japan).

### MLPA

The Multiplex Ligation-dependent Probe Amplification (MLPA) kit including the probe set corresponding to each exon of *WFS1* was purchased from MRC-Holland (Amsterdam, The Netherlands). The experiments were performed according to the manufacturer’s instructions [21]. Briefly, 200 ng of genomic DNA were denatured and hybridized to 1.5 µl of probemix along with 1.5 µl of SALSA MLPA buffer provided by MRC-Holland (Amsterdam, The Netherlands). The mix was incubated for 1 min at 95°C followed by 18-hr hybridization at 60°C. Next, the probes were ligated at 54°C for 15 min followed by an inactivation step of 5 min at 98°C. The PCR reaction was prepared using 10 µl of the ligation mix, PCR primers, polymerase, buffers and water, and the amplification was then performed under the following conditions: 95°C for 30 sec, 65°C for 90 sec, 30 cycles, followed by a final extension for 20 min at 72°C. Capillary electrophoresis was performed using an ABI 3130XL Genetic Analyzer (Applied Biosystems). A mix of 0.7 µl of PCR reaction solution, 8.9 µl of Hi-Di formamide, and 0.4 µl of GeneScan 600 LIZ Size Standard (Applied Biosystems) was denatured for 2 min at 85°C, spun down and loaded. ABI result files were normalized employing GeneMarker Software (Softgenetics, State College, PA, USA), using a predefined panel and imported to an Excel spread sheet for simple copy number calculations.

### Analysis of Genotype-Phenotype correlations for *WFS1* mutations

Patients were subdivided into three groups as follows. Group 1 includes individuals carrying nonsense mutations, frame-shift mutations and/or multiple amino acid insertion/deletions in both alleles. These mutations are likely to cause severe deteriorations or loss of WFS1 protein function. Group 2 includes individuals carrying missense mutations and/or single amino acid insertions in both alleles. These mutations have unknown functional consequences without experimental evidence, but most of them are likely to result in milder functional deteriorations than the mutations in group 1. Group 3 is defined as individuals with compound heterozygous mutations found in group 1 and group 2. Ages at the onset of diabetes mellitus and/or optic atrophy were compared among these groups.

### Statistical analysis

Analysis of variance (ANOVA) was used to compare data among the groups, with post-hoc analysis using Scheffé’s test. Contingency tables were used for categorical variables. The significance level was set at P<0.05. Statview 5TM software (SAS Institute, Inc., Cary, NC, USA) was used for all statistical analyses. Demographic data are presented as means ± SD.

## Results

### Clinical and genetic analyses

It has been recognized that diabetes mellitus is a hallmark manifestation and usually appears earliest among the components of WFS. Because diabetologists, endocrinologists or pediatricians were likely to be the caregivers first proposing the diagnosis, we exhaustively sought patients with the coexistence of diabetes mellitus and optic atrophy from diabetes referral centers and pediatric and adult diabetes clinics. Sixty-seven affected individuals were identified in this study. All had been diagnosed with both insulin requiring diabetes mellitus and optic atrophy prior to this study. As shown in Table 1, patients recruited for this study presented the following complications: diabetes insipidus in 37 patients (55%), hearing impairment in 50 (75%), renal tract abnormalities in 31 (46%), and neuro-psychiatric symptoms in 46 (69%). All four components of DIDMOAD were present in 33 patients (49%). Twenty-two patients (33%) had full-blown WFS presenting with 6 components.

**Table 1:** Prevalence of complications in 67 patients with WFS.

	Prevalence
Diabetes mellitus	67(100%)
Optic atrophy	67(100%)
Diabetes insipidus	37(55%)
Hearing impairment	50(75%)
Renal tract abnormalities	31(46%)
Neuropsychiatric illness	46(69%)

Further detailed clinical information on 40 patients from 30 unrelated families was obtained. Also, these patients were screened for *WFS1* mutations. No mitochondrial DNA mutations were found in those tested. Table 2 shows the clinical characteristics and family history with the genetic test results of each patient. The initial manifestation was diabetes mellitus at a median age of 8.7 years (9 m-30 years), followed by optic atrophy at a median age of 15.8 years (3–40 years). The subjects had been diagnosed based on the following clinical components; diabetes insipidus at a mean age of 17.2 (3–47) years, hearing impairment at a median age of 16.4 years (9 m-58 years), renal tract abnormalities at a median age of 20.2 years (birth-47 years), and neuro-psychiatric abnormalities at a median age of 24.4 (3–53) years. There were six patients in whom either optic atrophy (i.e. W13, W14, W17a, W27a, W27b) or diabetes insipidus (i.e. W7a) had been diagnosed prior to diabetes mellitus. In eight patients, other clinically apparent features, such as diabetes insipidus (i.e. W5, W7a and W7b), hearing impairment (i.e. W6d, W7a, W7b, W8, W21, W26, W27a) or psychiatric problems (i.e. W7a, W7b) had been diagnosed prior to optic atrophy. Various neurological symptoms were present in 7 patients, of whom W4, W26 and W27a had become symptomatic at a relatively early stage of the disease process. Seventeen patients had psychiatric problems. Notably, two affected siblings of W7 were diagnosed with attention deficit hyperactivity disorder as early as the onset of diabetes mellitus. In particular, morphological abnormalities of the cerebellum and brain stem had been documented by magnetic resonance imaging scans in 14 patients, 5 of whom had no neuro-psychiatric symptoms at the time of examination.

**Table 2:** Clinical characteristics and family history of each patient screened for *WFS1* mutations.

No.	Family	Sex	FH ofWFS	*WFS1*mutation	DM	OA	DI	D	Renal	Neuro	Psychiatric
1	W1	M	Y□	Y	3	4	−	6	−	−	−
2	W2	F	N	Y	9	32	32	41	41	+	N/A
3	W3	M	Y□	Y	6	11	47	19	47	−	−
4	W4	M	Y□	Y	4	12	−	−	−	12 (Dysarthria, Clumsiness)	−
5	W5	F	N	Y	7	10	7	17	20	43 (Brain atrophy, Dizziness)	Attempted suicide, Self injury
6	W6a	M	Y□	Y	5	9	12	12	11	26 (Brain atrophy)	−
7	W6b	M	Y□	Y	3	7	19	10	10	24 (Brain atrophy)	Depression
8	W6c	F	Y□	Y	4	6	11	7	7	18 (Brain atrophy)	Depression
9	W6d	F	Y□	Y	5	7	8	5	5	15 (Brain atrophy)	−
10	W7a	M	Y□	Y	5	9	4	5	N/A	N/A	5(ADHD)
11	W7b	M	Y□	Y	3	10	3	3	N/A	N/A	3(ADHD)
12	W8	M	N	Y	3	7	−	5	0	−	−
13	W9a	M	Y□	Y	3	5	+	+	+	16 (Brain atrophy)	Depression
14	W9b	M	Y□	Y	1.5	5	+	+	+	16 (Brain atrophy)	Depression
15	W9c	M	Y□	Y	5	+	N/A	+	N/A	N/A	N/A
16	W10a	F	Y	Y	17	18	−	−	−	N/A	Mental retardation
17	W10b	F	Y	Y	10	22	−	+	−	38 (Brain atrophy)	Depression
18	W11	F	N	Y	4	9	+	9	+	−	−
19	W12	M	N	Y	27	35	−	58	42	53 (Brain atrophy)	−
20	W13	F	N	Y	13	10	−	−	−	−	−
21	W14	F	N	Y	10	5	−	−	−	29 (Brain atrophy)	−
22	W15	F	N	Y	3	+	7	8	7	N/A	N/A
23	W16	F	N	Y	7	18	−	−	27	30 (Brain atrophy)	−
24	W17a	F	Y□	Y	23	14	−	−	+	27 (Brain atrophy, Nystagmus)	Mental retardation
25	W17b	F	Y□	Y	27	<29	−	−	25	29 (Brain atrophy, Nystagmus)	Mental retardation
26	W17c	F	Y□	Y	10	<30	−	−	+	30 (Brain atrophy, Nystagmus)	Mental retardation
27	W18	M	Y□	Y	1	<39	39	N/A	N/A	N/A	N/A
28	W19	M	N	Y§	3	26	−	−	−	−	−
29	W20	F	N	Y§	2	14	−	<41	−	N/A	Mental retardation
30	W21	M	N	N	30	40	−	0	−	−	−
31	W22	F	N	N	11	12	−	−	−	−	Mental retardation
32	W23	F	N	N	10	21	−	−	−	−	<22 (Depression, Self injury)
33	W24	F	N	N	+	+	N/A	+	N/A	N/A	N/A
34	W25	F	N	N	<22	<22	<22	N/A	N/A	N/A	N/A
35	W26	M	Y□	N	0(9 M)	7	−	0(9 M)	−	Spastic paraplegia	Mental retardation
36	W27a	M	Y	N	3	<3	−	<3	−	Nystagmus	Mental retardation
37	W27b	M	Y	N	7	<7	−	<11	−	−	−
38	W28	F	N	N	10	<27	−	−	−	−	−
39	W29	F	N	N	10	<29	−	−	−	−	−
40	W30	F	N	N	12	12	−	+	−	−	−

*M, male; F, female; DM, diabetes mellitus; OA, optic atrophy; D, deafness; DI, diabetes insipidus, numbers indicate age at onset in years; +, symptomatic with unknown onset age; −, asymptomatic, Y; Yes, N; No, N/A; not applicable, □; consanguineous marriage and affected siblings.

§ Individual with detectable *WFS1* mutation in single chromosome.

Twenty-seven patients (67.5%) from 18 families had mutations in both alleles, of whom 8 probands and 8 secondary patients were in consanguineous families (i.e., W1, W3, W4, W6, W7, W9, W17 and W18). Among these, 12 patients had been assessed for WFS due to the presence of *WFS1* mutations prior to this study. [5], [22], [23]. W19 and W20 had mutations in only one allele. On the other hand, no mutations in the exons or the exon/intron boundaries of the *WFS1* were found in 11 patients (27.5%) from 10 families, one of which was consanguineous (i.e. W26). Although the prevalence of both diabetes insipidus and renal tract involvement appeared to be relatively low in patients with intact *WFS1* alleles (Table 2), the ages at onset of diabetes mellitus and/or optic atrophy, representative clinical diagnostic features of this syndrome, were indistinguishable between the patients with recessive *WFS1* mutations and those without *WFS1* mutations (diabetes mellitus; 8.1±7.3 vs 11.6±8.6, p = 0.27, optic atrophy 14.5±10.5 vs 18±11.8, p = 0.43, respectively) (Table 3).

**Table 3:** Comparison of ages at onset of diabetes mellitus and optic atrophy in subjects with and without a mutated *WFS1* gene.

	*WFS1* mutation(+)	*WFS1* mutation(−)	
	(n = 27)	(n = 11)	*P*
Age at onset of DM	8.1±7.3	11.6±8.6	0.27
Age at onset of OA	14.5±10.5	18±11.8	0.43

*Data are expressed as means±SD.

### *WFS1* mutations

**Figure 1:**
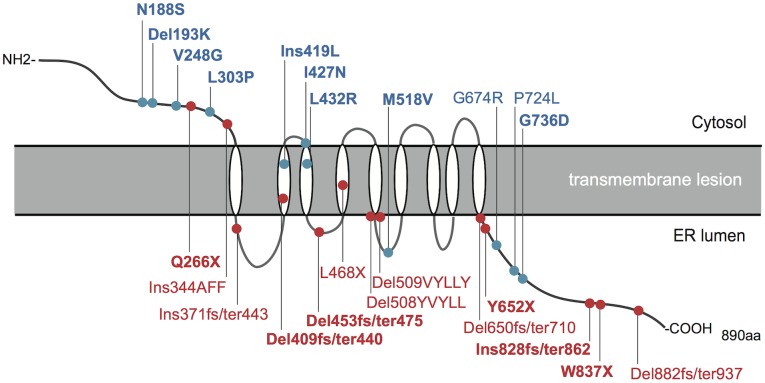
A schematic presentation of mutations affecting the WFS1 protein. The relative positions of WFS1 mutations within the putative WFS1 protein topology are indicated. Mutations are color-coded according to their mutation categories: mutations with predicted complete loss of function (red), mutations with predicted partial loss of function (blue). Novel mutations are indicated in bold type.

Table 4 shows the *WFS1* mutations with predicted amino acid changes in each patient. Of all the mutations presented, 16 have not previously been described elsewhere. In addition to the *WFS1* mutations associated with this syndrome, polymorphisms were found as listed in Table S3 in File S1. The mutational spectrum included missense, in-frame deletion, nonsense and smaller deletions and insertions, which were associated with a shift of the *WFS1* reading frame. Most mutations were located within exon 8, which encodes the putative transmembrane domain and COOH-terminal endoplasmic reticulum luminal domain of WFS1 protein (Figure 1). Four mutations were located in exon 5 and exon 7, both of which encode NH2-terminal cytosolic domains of the protein (Figure 1). We predicted deleterious effects of each missense mutation *in* *silico* by using *PolyPhen2* (http://genetics.bwh.harvard.edu/pph2) *and SIFT* (http://sift.jcvi.org/www/SIFT_enst_submit.html). All of the missense mutations but one (N188S) were predicted to be damaging by at least one prediction program (Table S4 in File S1).

**Table 4:** *WFS1* mutations in each patient with WFS.

Family	Mutationgroup	Exon	Nucleotidechanges	Amino acidchange	Type ofmutation	Firstdescription
W1	1	7	796C>T	**Q266X**	Nonsense	**Novel**
W2	1	8	1032–1033ins9	Ins344AFF	In-frame Insertion	Inukai et al.[2005]
W3	1	8	1109–1110ins5	Ins371fs/ter443	Frameshift	Nakamura et al.[2006]
W4	1	8	1228del(C)	**Del409fs/ter440**	Frameshift	**Novel**
W5	1	8	1401–1403del(GCT),1525–1539del15	L468X,Del509VYLLY	Nonsense+In-framedeletion	Fujimaki et al.[2011]+Chaussenot et al.[2011]
W6a	1	8	1515–1530del15	Del508YVYLL	In-frame deletion	Inoue et al.[1998]
W6b	1	8	1515–1530del15	Del508YVYLL	In-frame deletion	Inoue et al.[1998]
W6c	1	8	1515–1530del15	Del508YVYLL	In-frame deletion	Inoue et al.[1998]
W6d	1	8	1515–1530del15	Del508YVYLL	In-frame deletion	Inoue et al.[1998]
W7a	1	8	1956C>A	**Y652X**	Nonsense	**Novel**
W7b	1	8	1956C>A	**Y652X**	Nonsense	**Novel**
W8	1	8	2484ins(GA), 2510G>A	**Ins828fs/ter862,W837X**	Frameshift+Nonsense	**Novel+Novel**
W9a	1	8	2642del(TC)	Del882fs/ter937	Frameshift	Inoue et al.[1998]
W9b	1	8	2642del(TC)	Del882fs/ter937	Frameshift	Inoue et al.[1998]
W9c	1	8	2642del(TC)	Del882fs/ter937	Frameshift	Inoue et al.[1998]
W10a	3	5,8	577–579del(AAG),1949–1950del(AT)	**Del193K**,Del650fs/ter710	Deletion+Frameshift	**Novel+**Domenech et al.[2004] Chaussenot et ai.[2011]
W10b	3	5,8	577–579del(AAG),1949–1950del(AT)	**Del193K**,Del650fs/ter710	Deletion+Frameshift	**Novel+**Domenech et al.[2004] Chaussenot et ai.[2011]
W11	3	5,8	563A>G,1359del(C)	**N188S,Del453fs/ter475**	Missense+Frameshift	**Novel+Novel**
W12	2	5	577–579del(AAG)	**Del193K**	Deletion	**Novel**
W13	2	7,8	743T>G, 2020G>A	**V248G**,G674R	Missense+Missense	**Novel+**Gomez-Zaera et ai.[1999] Khanim et al.[2001]
W14	2	8	908T>C,1254ins(TCT)	**L303P,Ins419L**	Missense+In-frameInsertion	**Novel+Novel**
W15	2	8	1280T>A	**I427N**	Missense	**Novel**
W16	2	8	1295T>G,1552A>G	**L432R,M518V**	Missense+Missense	**Novel+Novel**
W17a	2	8	2171C>T	P724L	Missense	Inoue et al.[1998]
W17b	2	8	2171C>T	P724L	Missense	Inoue et al.[1998]
W17c	2	8	2171C>T	P724L	Missense	Inoue et al.[1998]
W18	2	8	2207G>A	**G736D**	Missense	**Novel**
W19§	N/A	8	1228del(C)	**Del409fs/ter440(hetero)**	Frameshift	**Novel**
W20§	N/A	8	2425G>A	**E809K(hetero)**	Missense	**Novel**

*Novel mutations are indicated in boldface.

§ Individual with detectable *WFS1* mutation in single chromosome.

Two mutations were found in only one chromosome (i.e. W19, W20). One of them, 1228del(C); del409fs/ter440 in W19, was also present homozygously in W4. Family W19 was not reported to be related to family W4. Patient W19 had no siblings and the parents had no symptoms of WFS. The other, 2425G>A; E809K in W20, has not previously been reported and was not found in 100 healthy Japanese controls, suggesting it to be a rare mutation. DNA from the father was the only sample available for examination of this patient’s relatives. However, the same mutation was not found (data not shown). Family W4 was consanguineous. The parents were not reported to be affected by WFS. Patient W20 had no siblings and the parents had no symptoms of WFS. In addition to these cases with a single mutated chromosome, there were 11 patients in which no *WFS1* mutations were identified. To assess whether complete or partial gene deletions of the *WFS1* region exist in such cases, the copy number variations of each exon of *WFS1* in genomic sequences were tested using MLPA in W19, W20, W21, W22, W23, W24 and W25. However, the copy number of each exon in the tested patients did not differ from those in the healthy control subject (Figure S1 in File S1). Furthermore, no mutations of the *WFS2* gene were identified by PCR direct sequencing in these patients.

### Genotype-phenotype correlation of *WFS1*

**Figure 2:**
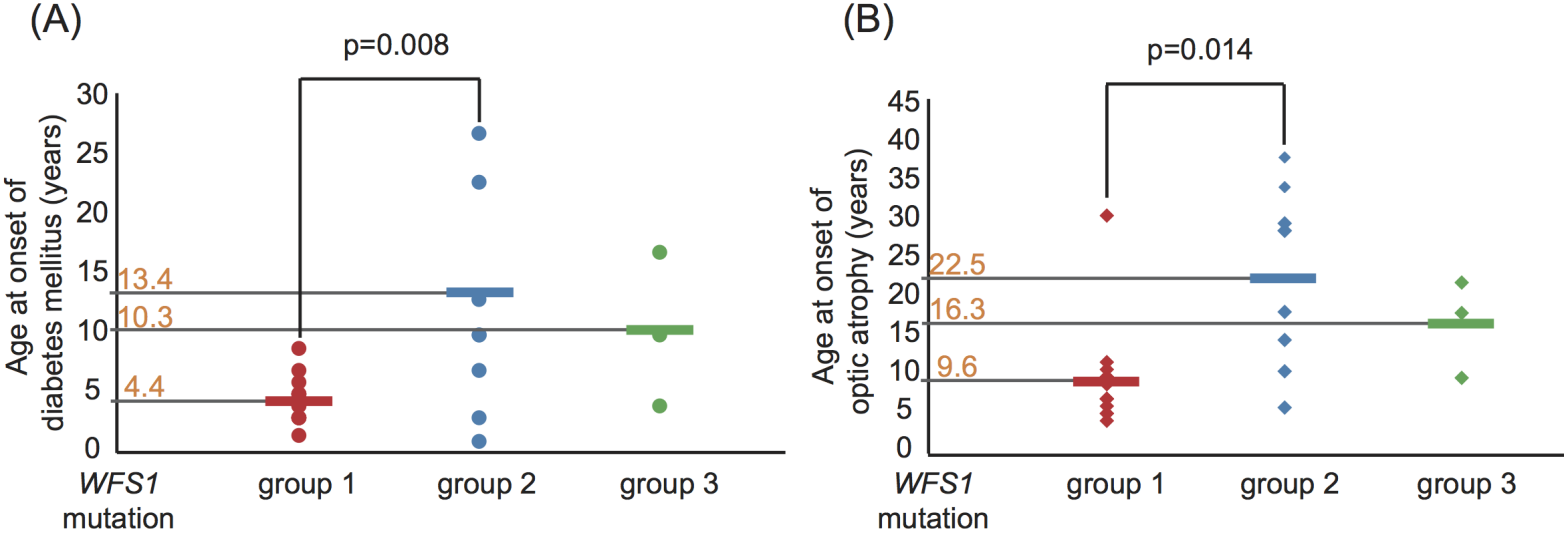
Analysis of genotype-phenotype correlations for *WFS1* mutations. Ages at onset of both diabetes mellitus (A) and optic atrophy (B) in each patient in the three groups are shown graphically with the mean age indicated on the vertical axis. Patients are color-coated according to the mutation categories: group 1 (n = 15) in red, group 2 (n = 9) in blue and group 3 (n = 3) in green. The differences between group 1 and group 2 were statistically significant: diabetes mellitus 4.4±1.9 years vs. 13.4±9.9 years, *p* = 0.008, and optic atrophy 9.6±6.9 years vs. 22.5±12.4 years, *p* = 0.014, respectively. Data are expressed as means ± SD.

To assess genotype-phenotype correlations for *WFS1*, patients were divided into three groups according to the predicted functional consequences of each mutation for WFS1 protein function (Table 4, Figure 1). The ages at onset of diabetes mellitus and/or optic atrophy were compared among the groups. Both features emerged significantly earlier in group 1 than in group 2, (diabetes mellitus; 4.4±1.9 years vs. 13.4±9.9 years, p = 0.008, optic atrophy; 9.6±6.9 years vs. 22.5±12.4 years, p = 0.014) (Figures 2A and B). Group 3 showed intermediate values. The ages at onset of each feature were variable in group 2 as compared to group 1. Among group 2 subjects, consanguineous affected siblings of W17 with homozygous P724L were previously described as presenting with late onsets of both diabetes mellitus and optic atrophy with the absence of diabetes insipidus [5]. In addition, W12 with a homozygous K193del in group 3 presented with apparently milder phenotypic features (Table 2). Moreover, despite the presence of a predicted complete loss of function mutation in another allele, affected siblings of W10 with compound heterozygous K193del had adolescent-onset diabetes mellitus with neither diabetes insipidus nor hearing impairment.

### Prevalence studies

Thirty patients were alive in the selected regions in 2010. There were 8 patients in the Tokyo metropolitan area, 12 in the Osaka metropolitan area and 10 in the Tohoku region. The respective estimated numbers of living cases were 11.2, 16.8 and 14 (see Patients and Methods section). The total population at mid-year 2010 was 13,159,388 in the Tokyo metropolitan area, 8,865,245 in the Osaka metropolitan area, and 9,335,636 in the Tohoku region. We thus estimated a mean prevalence in these three regions of 1.415 cases per million people, or one in 710,000 of the total population.

## Discussion

WFS is a rare hereditary recessive disorder associated with multiple clinical manifestations. Comprehensive, standardized phenotyping and assessment of the contributions of *WFS1* mutations to clinical features of patients with WFS have thus far been limited. The current study documented the following observations; 1) WFS was confirmed to be rare with the prevalence being 1/710,000 in the Japanese population. 2) The order in which symptoms emerge in the process of WFS development is variable. 3) When WFS is defined by the minimum ascertainment criteria of the coexistence of early onset diabetes and insidious bilateral optic atrophy, approximately 70% of affected Japanese patients presented with recessive *WFS1* mutations, and the remaining patients had either a single mutated allele or intact alleles. 4) Genotypic class-phenotype correlations may exist, as demonstrated by age at onset of diabetes mellitus and optic atrophy. These lines of evidence provide additional insights into the clinical features and genetics of this complex syndrome, which could facilitate diagnosing WFS and ultimately be useful for the development of therapeutic interventions.

The prevalence of WFS in the Japanese population was very close to that reported in the UK (1/710,000 vs. 1/770,000) [2]. The proportion of our patients having the four components of DIDMOAD was comparable to that in former studies using similar patient identification criteria (49% vs. 53% in the UK, 58% in Lebanon) [2], [16]. Diabetes mellitus and optic atrophy are the two most common initial manifestations in Japanese patients with WFS. However, either optic atrophy or diabetes insipidus emerged prior to the onset of diabetes mellitus in six patients. Furthermore, optic atrophy does not always predate other clinically apparent features, such as diabetes insipidus, hearing impairment or psychiatric problems. Therefore, the observations made in our limited sample suggested that the order in which symptoms emerge in the course of WFS is difficult to discern. Based on the variable clinical courses of patients with WFS documented in our and another study [4], the diagnosis must be considered when patients present with two or more of any of the DIDMOAD features and neurological-psychiatric problems.

Earlier diagnosis of this disease is anticipated to improve quality of life for patients with WFS by allowing earlier interventions aimed at managing the clinical features of this syndrome. Diabetologists, as well as adult and pediatric endocrinologists, pediatricians and ophthalmologists, are likely to have the first opportunity to recognize WFS after the diagnosis of an initial manifestation, and school health screening programs may also provide an opportunity to identify vision defects, hearing impairment, and abnormal urinalysis results. However, a significant delay in the recognition of WFS may occur in some patients, and this would presumably impact their quality of life adversely by prolonging the period of untreated associated symptoms. A recent report revealed that color blindness and loss of olfaction are common even in the youngest subjects with WFS [24]. These features are likely to be rather easily screened for at an outpatient clinic and therefore may facilitate earlier diagnosis of WFS. Thus, the specificity of such sensorial symptoms in WFS requires further evaluation in larger cohorts. On the other hand, the genetic test for *WFS1* may allow early diagnosis even before the onset of diabetes mellitus or diabetes insipidus in patients with hearing loss, vision defects, family history, and so on, if there is an index of suspicion for this disorder.

In the current study, genetic screening for *WFS1* mutations confirmed this to be a major gene responsible for WFS, and that genetic heterogeneity of this disease may exist in Japanese patients with WFS. This genetic evidence raised the possibility of mutations existing in the other genes. However, mutations in the promoter or other regulatory regions of *WFS1* could not be ruled out, because we sequenced only 113 bp upstream from exon 1 and the exon-intron boundaries. In fact, mutations were identified only in one chromosome in two patients. In addition to these limitations, the existence of large rearrangements or deletions of the *WFS1* locus might be considered, although MLPA analyses did not detect such abnormalities in our limited subject sample (Figure S1 in File S1).

The ages at onset of diabetes mellitus and/or optic atrophy were not associated with the presence or the absence of *WFS1* mutations in our patients (Table 3). However, longitudinal follow-up of larger cohorts will be necessary to fully address whether the presence or the absence of *WFS1* mutations predicts the long-term course of WFS.

The broad spectrum of *WFS1* mutations identified might represent different effects on WFS1 protein. The genotypic class-phenotype correlation performed in previous studies [18], [20] showed that differences in ages at the onset of diabetes mellitus and/or optic atrophy correlated with genotypic classifications (Figure 1 and 2). Taking another view of such analyses, disease onset and progression were relatively similar in patients in whom WFS1 function was thought to be virtually abolished (Figure S2 in File S1). By contrast, the patients with *WFS1* variations associated with either a single amino acid substitution or deletion presented with variable disease progressions (Figure S2 in File S1). Some had apparently milder phenotypes than those with other mutations, indicating differences in the effects of such mutations on WFS1 protein functions. Although the effects of such variations on functions, cellular localization and protein expression remain unknown, these observations may provide an opportunity to determine unknown functional domains disrupted specifically by mutations in the WFS1 protein.

Because insulin secreting beta cells are thought to be the most susceptible tissue in WFS, numerous investigations of *WFS1* biology have focused on pancreatic beta cells. WFS1 deficient beta cells have been shown to be susceptible to ER stress-induced-apoptosis [7], [8], [25] and to have hormone secretion defects [7], [10]. Furthermore, *WFS1* is one of the genes reproducibly shown to be associated with Type 2 diabetes [26], [27], [28], [29]. Among identified *WFS1* SNPs (Table S3 in File S1), rs734312 (H611R) was shown to be associated with type 2 diabetes, with the minor allele conveying protective effects [26], [28]. These lines of genetic evidence indicate that WFS may be an unusual and extreme model of Type 2-like diabetes mellitus with a monogenic cause. This characteristic may present an opportunity for testing diabetes therapies in a monogenic setting or further delineating the mechanism of beta cell failure causing diabetes progression.

In conclusion, our study is one of the largest to date concerning WFS. Our data provide additional insights into the clinical features of this disease and allow a better understanding of the underlying genetic mechanisms. The information obtained in this study may facilitate future genetic and molecular investigations leading to a clearer understanding of the pathophysiology of WFS and ultimately to improvements in prevention strategies as well as treatments for this devastating disease.

## Supporting Information

File S1 **This file contains Supporting Figures and Tables.** Figure S1. Multiplex Ligation–dependent Probe Amplification (MLPA) analysis. MLPA was performed in seven patients without recessive *WFS1* mutations. The luminescence peak area of WFS1 exons measured in each patient relative to that of a healthy control subject was expressed graphically. Figure S2. Ages at onset of both diabetes mellitus and optic atrophy in each patient in three genotypic classes. Ages at onset of both diabetes mellitus and optic atrophy are shown in the same graph. Patients are color-coded according to their mutation categories. Group 1 (severe) is in red, group 2 (mild) in blue, and group 3 (intermediate) in green. Table S1. PCR and sequencing primers for *WFS1.* Table S2. DNA probes corresponding to the indicated mutations in *WFS1* for TaqMan PCR analysis. Table S3. The list of detectable polymorphisms in the *WFS1* gene. Table S4. *In silico* scores and predictions of deleterious effects of missense mutations on the WFS1 protein.
(PDF)
