# Supplementary material for: Wolfram Syndrome in the Japanese Population; Molecular Analysis of WFS1 Gene and Characterization of Clinical Features
Source: PLoS One. 2014 Sep 11;9(9):e106906. doi: 10.1371/journal.pone.0106906 (PMC4161373; doi:10.1371/journal.pone.0106906)
Supplement: File S1 — This file contains Supporting Figures and Tables. Figure S1. Multiplex Ligation–dependent Probe Amplification (MLPA) analysis. MLPA was performed in seven patients without recessive WFS1 mutations. The luminescence peak area of WFS1 exons measured in each patient relative to that of a healthy control subject was expressed graphically. Figure S2. Ages at onset of both diabetes mellitus and optic atrophy in each patient in three genotypic classes. Ages at onset of both diabetes mellitus and optic atrophy are shown in the same graph. Patients are color-coded according to their mutation categories. Group 1 (severe) is in red, group 2 (mild) in blue, and group 3 (intermediate) in green. Table S1. PCR and sequencing primers for WFS1. Table S2. DNA probes corresponding to the indicated mutations in WFS1 for TaqMan PCR analysis. Table S3. The list of detectable polymorphisms in the WFS1 gene. Table S4. In silico scores and predictions of deleterious effects of missense mutations on the WFS1 protein. (PDF) [file pone.0106906.s001.pdf]

Figure S1. Multiplex Ligation–dependent Probe Amplification (MLPA) analysis.

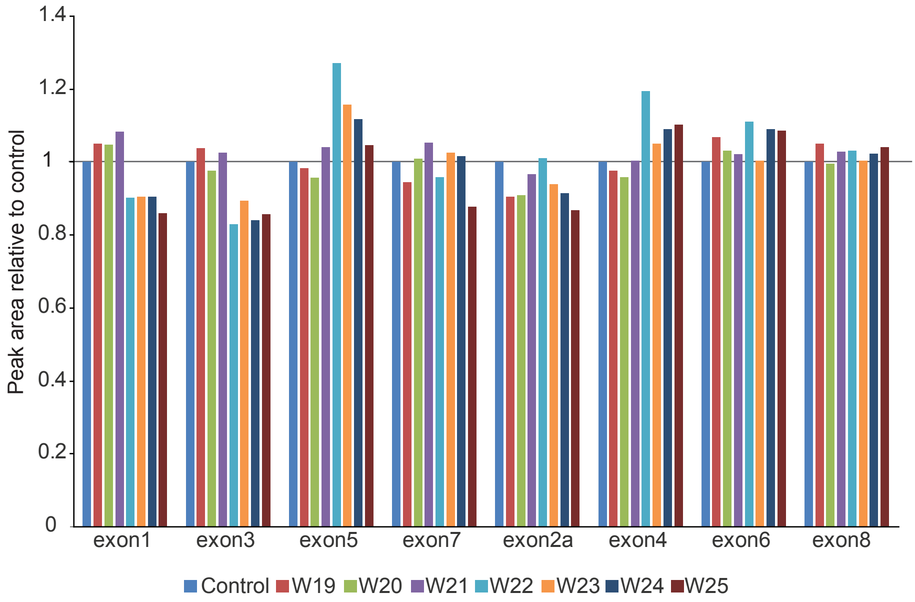

Figure S2. Age at onset of both DM and OA in each patient in three genotypic classes.

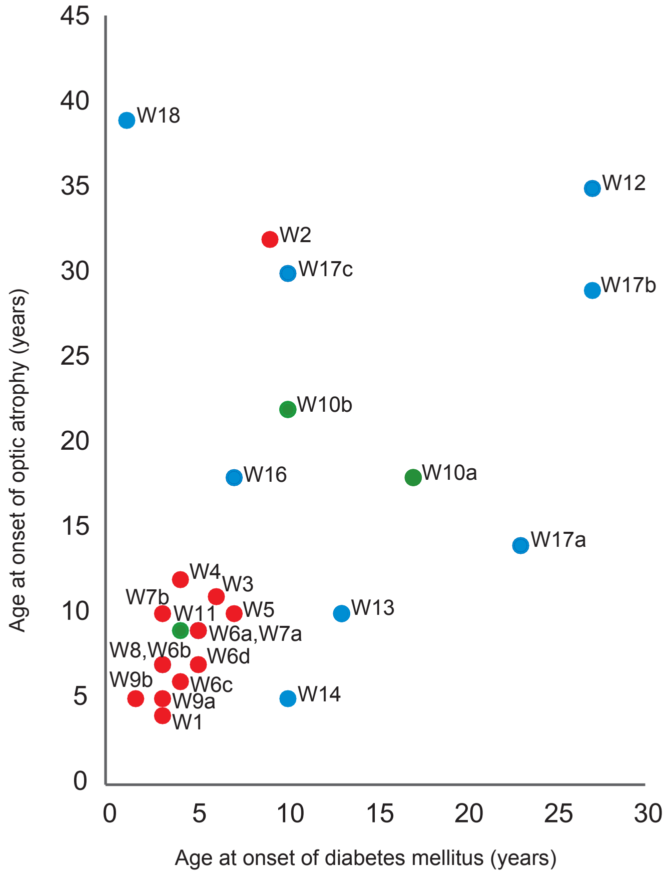

Table S1. PCR and sequencing primers for *WFS1* gene

Exon	Forward primer(5'-3')	Reverse primer(5'-3')
1	TGGAGTGATTGGCGGCTACA	AGGCCCGAGGAGGACAGTGC
2	CTGTCTCCAGCAGACACTAAGTGCCA	CCCACCCAGCTATCCCTGAACATCC
3	CTGAAGACCCTCATGCCTTG	ACACTTCTCTGTGGGCTGTG
4	TCGGAGAATCTGGAGGCTGA	CATTACAAGCTGCTCAACCC
5	CGAAAGCCTTCCAGGCAGAG	CTATGGGAAGGTCCTGGCTC
6	CTAGGAACAGTGCGCCAGTT	ATGGAGTCGCACAGGAAGGA
7	GCCCATGCTGTTTTCTCTCA	CCGAGGACACATCCTTATGA
8(a)	GCGTGAGATGGGAGCAGTGG	CTGGCGATGGGGAAGGAGAA
8(b)	TCCGCACCCTCACCGACCTG	CACACCAGGTAGGGCACAAG
8(c)	CCTGGTCGTCCTCAATGTCA	CATAGAACCAGCAGAACAGC
8(d)	CCGTGGCGGTCTGTAGTGTGC	CCCACGGTAATCTCAAACTT
8(e)	CTGGATGCGCTGCCTCTACG	CATGGCAAGATGCACTGGAAGC

Table S2. DNA probes corresponding to the indicated mutations in *WFS1* for TaqMan PCR analysis

Mutation	Forward primer (5'-3')	Reverse primer (5'-3')	Probe-VIC	Probe-FAM	n
E809K	GACGTCACCAAGGACATCGT	CTCCAGGATGGTGCTGAACTC	CCAGCAGCGAGTTCA	CAGCAGCAAGTTCA	100
Del193K	TGGTCATGTACTGGAAGCTCAAC	TTGACCTGGCCGACATTCTC	CAAGAAGAAGAAGCAGGTG	CCAAGAAGAAGCAGGTG	100
V248G	AAGAACTACATCGCGCTGGAT	CGCCCTTGGCGTACTTCTTAG	TGATCTCCACAAAGTC	ATCTCCCCAAAGTC	183

Table S3. The list of detectable polymorphism in *WFS1*.

Exon	Nucleotide change	Aminoacid change	1000 Genomes
8	1234G>C	p.V412L	rs149865710
8	1367G>A	p.R456H	rs1801208
8	1726G>A	p.G576S	rs1805069
8	1832G>A	p.R611H	rs734312
8	2158A>G	p.I720V	rs1805070
8	2369C>T	p.S790L	not reported
8	2469C>T	p.I823I	rs181025
8	2596G>A	p.D866N	rs3821945

Table S4. *In Silico* scores and predictions of functional consequences of non-synonymous variants of *WFS1*.

Programs	PolyPhen-2	SIFT
	HumVar Score<br>Prediction	Score<br>Prediction
p.N188S	0.166<br>Benign	0.55<br>Tolerated
p.V248G	0.975<br>Probably damaging	0<br>Damaging
p.L303P	0.998<br>Probably damaging	0<br>Damaging
p.I427N	0.809<br>Possibly damaging	0<br>Damaging
p.L432R	0.999<br>Probably damaging	0<br>Damaging
p.M518V	0.526<br>Possibly damaging	0.18<br>Tolerated
p.G674R	0.999<br>Probably damaging	0.03<br>Damaging
p.P724L	1<br>Probably damaging	0<br>Damaging
p.G736D	1<br>Probably damaging	0<br>Damaging
p.E809K	0.934<br>Probably damaging	0<br>Damaging

Polyphen2 computes both HumDiv score and HumVar score to predict deleterious effects of amino acid substitutions. HumVar is preferred model for diagnosis of Mendelian disease. Regarding SIFT, amino acid substitutions with scores <0.05 are predicted to be deleterious.
